# Successful identification of a predictive biomarker for lymph node metastasis in colorectal cancer using a proteomic approach

**DOI:** 10.18632/oncotarget.22149

**Published:** 2017-10-30

**Authors:** Koichiro Mori, Yuji Toiyama, Kohei Otake, Shozo Ide, Hiroki Imaoka, Masato Okigami, Yoshinaga Okugawa, Hiroyuki Fujikawa, Susumu Saigusa, Junichiro Hiro, Minako Kobayashi, Masaki Ohi, Koji Tanaka, Yasuhiro Inoue, Yuhko Kobayashi, Yasuhiko Mohri, Issei Kobayashi, Ajay Goel, Masato Kusunoki

**Affiliations:** ^1^ Department of Gastrointestinal and Pediatric Surgery, Mie University Graduate School of Medicine, Tsu, Mie, Japan; ^2^ Center for Molecular Biology and Genetics, Mie University, Mie, Japan; ^3^ Center for Gastrointestinal Research & Center for Epigenetics, Cancer Prevention and Cancer Genomics, Baylor Scott & White Research Institute and Charles A Sammons Cancer Center, Baylor University Medical Center, Dallas, TX USA

**Keywords:** iTRAQ-based quantitative proteomic analysis, ezrin, lymph node metastasis, colorectal cancer, biomarker

## Abstract

Colorectal cancer (CRC)-associated mortality is primarily caused by lymph node (LN) and distant metastasis, highlighting the need for biomarkers that predict LN metastasis and facilitate better therapeutic strategies. We used an Isobaric Tags for Relative and Absolute Quantification (iTRAQ)-based comparative proteomics approach to identify novel biomarkers for predicting LN metastasis in CRC patients. We analyzed five paired samples of CRC with or without LN metastasis, adjacent normal mucosa, and normal colon mucosa, and differentially expressed proteins were identified and subsequently validated at the protein and/or mRNA levels by immunohistochemistry and qRT-PCR, respectively. We identified 55 proteins specifically associated with LN metastasis, from which we selected ezrin for further analysis and functional assessment. Expression of ezrin at both the protein and mRNA levels was significantly higher in CRC tissues than in adjacent normal colonic mucosa. In univariate analysis, high ezrin expression was significantly associated with tumor progression and poor prognosis, which was consistent with our *in vitro* findings that ezrin promotes the metastatic capacity of CRC cells by enabling cell invasion and migration. In multivariate analysis, high levels of ezrin protein and mRNA in CRC samples were independent predictors of LN metastasis. Our data thus identify ezrin as a novel protein and mRNA biomarker for predicting LN metastasis in CRC patients.

## INTRODUCTION

Colorectal cancer (CRC) is one of the most common malignancies worldwide and is a major cause of cancer-related deaths [1]. One important factor that contributes to the poor prognosis for CRC patients is the rapid occurrence of lymph node and distant metastasis, which reduces the 5-year survival rate to 69.2% upon lymph node (LN) metastasis [1]. The treatment of CRC has evolved towards a multimodality management approach that includes surgical resection, chemotherapy, and radiotherapy [2]. However, the development of tools for accurate preoperative detection of LN metastasis in CRC could play an important role in therapeutic decision-making, such as the potential benefit of neoadjuvant and/or adjuvant chemotherapy. In contrast, the absence of LN metastasis in preoperative samples might suggest a more conservative approach to keep bowel resection to a minimum.

The American Joint Committee on Cancer TNM staging is currently the most accepted prognostic criteria for CRC patients. Accurate preoperative staging of CRC is essential for deciding the most pertinent and effective treatment strategies. Noninvasive imaging modalities frequently used for the preoperative diagnosis of LN metastasis in CRC patients include computed tomography (CT), magnetic resonance imaging (MRI), endorectal ultrasonography, and positron emission tomography/CT [3, 4]. However, these imaging approaches are generally unreliable and inadequate at identifying LN metastasis (accuracy rates: CT 22–73%, MRI 39–95%, EUS 62–83%) [4]. Meta-analysis of common histopathological findings have revealed that lymphatic invasion, tumor depth, tumor differentiation, and tumor budding are predictive factors for LN metastasis [5]. In addition, several preoperative biomarkers have the potential to act as complementary tools to improve LN metastasis classification in CRC patients, which include tumor markers such as CA19-9, c-reactive protein, neutrophil-to-lymphocyte ratio and platelet-to-lymphocyte ratio [6-10]. However, none of these markers are currently recommended as routine screening tools because of their inherently low sensitivity and specificity.

Biomarker discovery for CRC has mainly focused on the identification of microRNAs, long non-coding RNAs, and methylated DNA in colonic tissues. Proteomics approaches are powerful tools for mapping the proteomes of tissues, cells, and organisms in the quest for new disease biomarkers [11]. A number of quantitative proteomics approaches have been widely used for biomarker discovery, including surface-enhanced laser desorption/ionization, two-dimensional gel electrophoresis–mass spectrometry (MS), and isobaric tags for relative and absolute quantitation (iTRAQ). This latter technique was developed to quantify relative changes in protein abundance in various biological samples with high accuracy and reproducibility. The technique involves differentially labeling fragmented peptides from distinct proteomes, such as normal versus malignant tissues, and then pooling them for liquid chromatography (LC)-MS/MS. The individual tags give rise to unique ions that allow the peptides originating in each starting proteome to be quantified and identified within the same sample [12]. The use of iTRAQ reagents with four or eight tags allows for multiplexing of multiple samples in one experiment.

The aim of this study was to identify novel predictive biomarkers of LN metastasis in CRC by using an iTRAQ-based comparative proteomics approach. Herein, we identified and also validated candidate biomarkers by examining protein expression and/or mRNA levels in CRC and adjacent normal mucosae by immunohistochemical staining and quantitative real-time PCR analysis, respectively. Finally, we selected the most promising candidate proteins and analyzed its oncogenic properties through functional studies.

## RESULTS

### Protein identification and quantification

**Figure 1:**
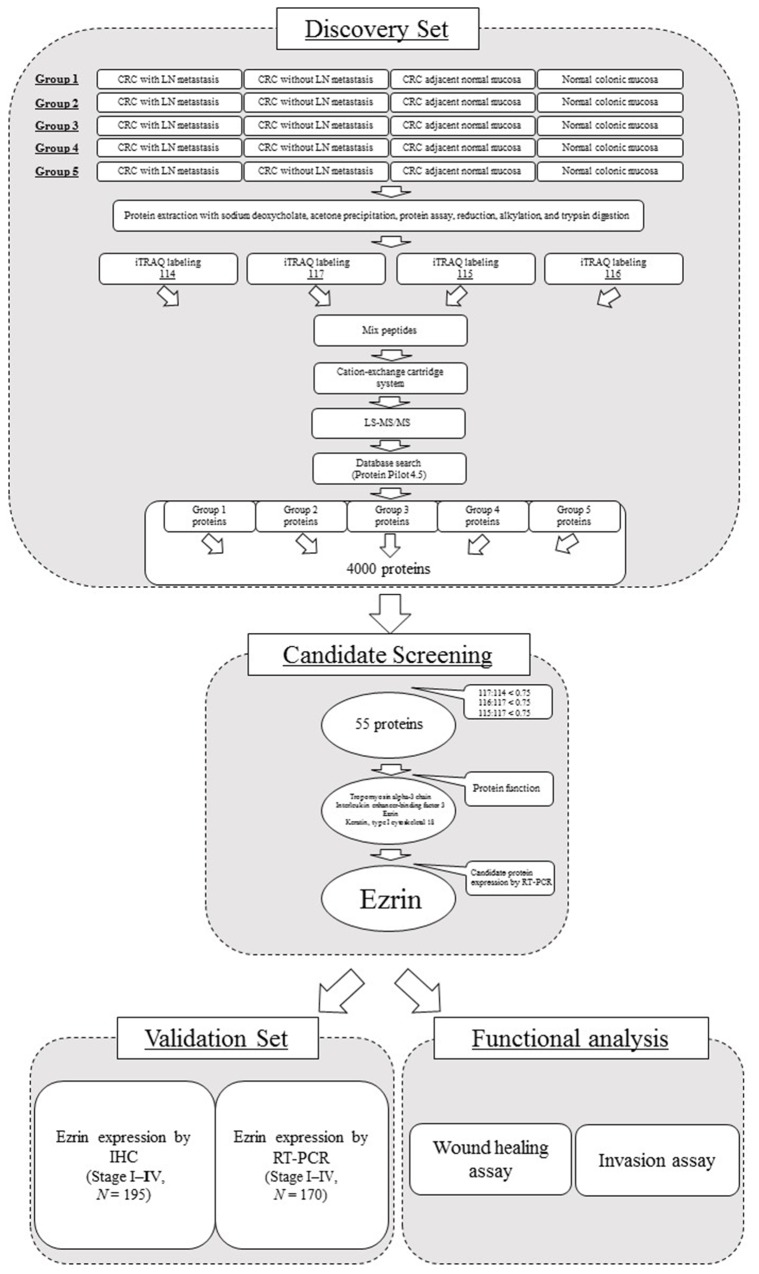
Summary of the workflow used for identification, validation, and functional analysis of a biomarker for CRC with LN metastasis

A summary of the workflow for iTRAQ-based quantification of differentially expressed proteins is shown in Figure 1. The four samples compared were CRC with and without LN metastasis, adjacent normal colonic mucosa, and normal colonic mucosa (labeled as 114, 117, 115, and 116 iTRAQ reagents, respectively). Following completion of the experimental protocol and data analysis with ProteinPilot software, we identified a total of 4000 proteins differentially expressed between the four samples. From these, we selected 55 proteins by using the ratio of 117:114 was less than 0.75, and both 116:117 and 115:117 ratios were also less than 0.75 per 117:114 to candidate markers for CRC with LN metastasis *versus* CRC without LN metastasis *versus* CRC adjacent normal colonic mucosa, and normal colonic mucosa (detailed in Supplementary Table 1). To obtain a better understanding of the molecular and functional characteristics of the 55 proteins, we classified them according to their molecular function (Supplementary Figure 2), cellular component (Supplementary Figure 2), and biological process (Supplementary Figure 3) using PANTHER (Protein Analysis through Evolutionary Relationship) Classification System (http://www.pantherdb.org). Finally, we selected 4 proteins from the category of developmental process (Supplementary Figure 3), which includes important metastatic processes such as the epithelial–mesenchymal transition [13].

**Figure 2:**
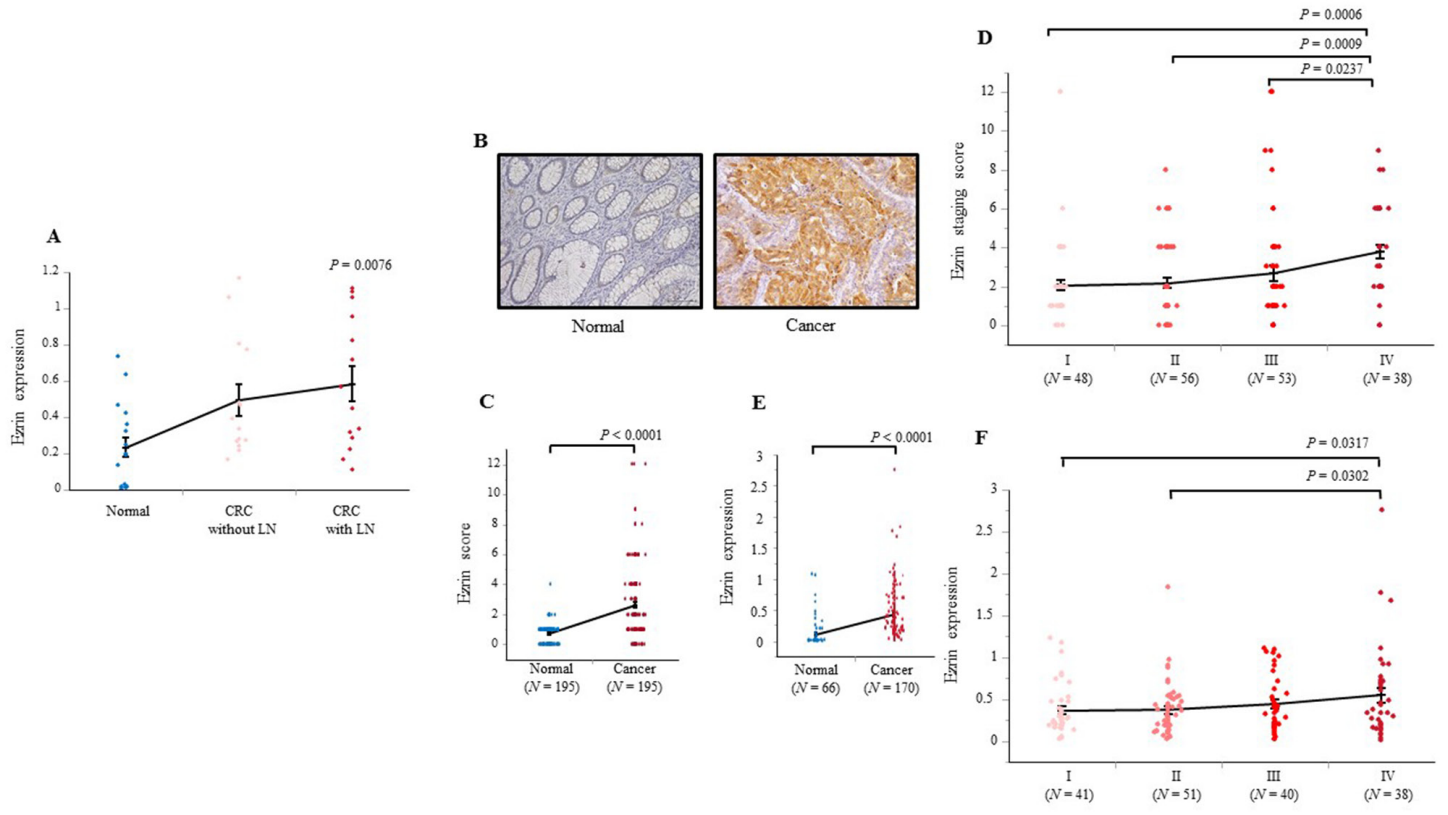
Ezrin protein and mRNA expression in screening and validation sets of colonic tissue **(A)** Ezrin mRNA levels in a subset of specimens from normal mucosa (*N* = 17), CRC without LN metastasis (*N* = 14), and CRC with LN metastasis (*N* = 14). **(B)** Representative photomicrographs showing IHC analysis of ezrin expression in adjacent normal mucosa and CRC. **(C)** IHC scores for ezrin expression in CRC and adjacent normal mucosal samples from 195 patients. **(D)** IHC scores for ezrin protein expression in 195 CRC samples subdivided by TNM staging. **(E)** Ezrin mRNA expression levels in colon samples from 66 healthy patients and 170 patients with CRC. **(F)** Ezrin mRNA levels in specimens from 170 CRC patients subdivided by TNM staging. Bars represent the SEM; the internal horizontal line indicates the median value. Statistical analysis was performed using Wilcoxon, Kruskal–Wallis, and Student’s t tests. Images were captured at ×100 magnification.

Of particular interest, we noted four prominent proteins associated with metastasis in the “development process” group; namely, tropomyosin alpha-3 chain, interleukin enhancer-binding factor 3, ezrin, and keratin, type I cytoskeletal 18 (Supplementary Figure 3). Finally, we selected ezrin as the most promising candidate predictive marker because its mRNA levels were higher in CRC with LN metastasis than in CRC without LN metastasis, and in turn, the expression in CRC without LN was higher than in normal colonic mucosa (*P* = 0.0076; Figure 2A). In contrast, levels of tropomyosin alpha-3 chain, interleukin enhancer-binding factor 3, and keratin, type I cytoskeletal 18 mRNA did not differentiate between normal mucosa, CRC without LN metastasis, and CRC with LN metastasis (Supplementary Figure 4).

### Association between ezrin expression and clinicopathological features in CRC

To assess the associations between ezrin expression and various clinicopathological features of CRC patients, we divided the 195 samples in Cohort 1 and 170 samples in Cohort 2 into two groups based on high or low ezrin expression (Table 1). The high/low cutoff values were determined by receiver operating characteristic (ROC) analysis for LN metastasis (Cohort 1 cutoff = 2, Cohort 2 cutoff = 0.71). IHC analysis was performed on Cohort 1 samples to investigate the cellular distribution of ezrin protein and to evaluate the clinical significance of ezrin expression in CRC. Ezrin expression was detected mainly in the cytoplasm in CRC specimens, whereas no staining was observed in adjacent normal mucosa (Figure 2B). Quantification of the intensity and extent of ezrin staining revealed that it was expressed at significantly higher levels in CRC than in normal mucosa (*P* < 0.0001; Figure 2C) and at significantly higher levels in stage IV CRC than in stage I, II, or III CRC (*P* = 0.0006, *P* = 0.0009, *P* = 0.0237, respectively; Figure 2D). Of the 195 CRC samples in Cohort 1, ezrin protein expression was high (score >2) in 57.4% (112/195) of cases and low in 42.6% (83/195) of cases. High ezrin staining was significantly associated with undifferentiated histology (*P* = 0.0158), LN metastasis (*P* = 0.0009), hepatic metastasis (*P* = 0.0019), peritoneal metastasis (*P* = 0.0111), distant metastasis (*P* = 0.0002), and TNM stage progression (*P* < 0.0001, Kruskal–Wallis test; Table 1). Cohort 2 samples were subjected to analysis of ezrin mRNA expression levels, which were significantly higher in CRC (*N* = 170) than in normal colonic mucosa (*N* = 66; *P* < 0.0001; Figure 2E) and significantly higher in stage IV CRC than in stage I or II CRC (*P* = 0.0317 and *P* = 0.0302, respectively; Figure 2F). Of the 170 CRC samples in Cohort 2, ezrin gene expression was high (>0.71) in 17.6% (30/170) of cases and low in 82.4% (140/170) of cases. High ezrin mRNA expression was significantly associated with undifferentiated histology (*P* = 0.0405), LN metastasis (*P* = 0.042), and distant metastasis (*P* = 0.0302) (Table 1).

**Table 1:** Correlations between expression of ezrin and clinicopathological features in CRC

Cohort 1: protein analysis^a^	*N*	Ezrin protein expression^b^		*P* value	Cohort 2: mRNA analysis^c^	*N*	Ezrin mRNA expression^b^		*P* value
Variables		high (*N* = 112)	low (*N* = 83)		Variables		high *(N* = 30)	low (*N* = 140)	
Age (years)					Age (years)				
<67	83	44	39	0.432	<67	72	17	55	0.0804
≥67	112	53	59		≥67	98	13	85	
Sex					Sex				
male	113	68	45	0.3635	male	99	15	84	0.3135
female	82	44	38		female	71	15	56	
Histology					Histology				
undifferentiated	15	13	2	**0.0158**^d^	undifferentiated	13	5	8	**0.0405**
differentiated	176	96	80		differentiated	157	25	132	
Tumor size (mm)					Tumor size (mm)				
<41	83	53	30	0.1186	<45	90	29	61	0.1661
≥41	112	59	53		≥45	80	34	46	
T classification					T classification				
Tis, T1, T2	70	36	34	0.2042	Tis, T1, T2	48	9	39	0.813
T3, T4	125	76	49		T3, T4	122	21	101	
Lymph node metastasis					Lymph node metastasis				
present	82	56	26	**0.009**	present	63	16	47	**0.042**
absent	113	56	57		absent	107	14	93	
Lymphatic invasion					Lymphatic invasion				
present	140	82	58	0.6089	present	149	26	123	0.8573
absent	55	30	25		absent	21	4	17	
Venous invasion					Venous invasion				
present	67	40	27	0.6434	present	133	24	109	0.7963
absent	128	72	56		absent	37	6	31	
Hepatic metastasis					Hepatic metastasis				
present	20	18	2	**0.0019**	present	26	7	19	0.1776
absent	175	94	81		absent	144	23	121	
Peritoneal metastasis					Peritoneal metastasis				
present	16	14	2	**0.0111**	present	4	0	4	0.3488
absent	179	98	81		absent	166	30	136	
Distant metastasis					Distant metastasis				
present	25	23	2	**0.0002**	present	20	7	13	**0.0302**
absent	170	89	81		absent	150	23	127	
Stage					Stage				
I	48	22	26	**<0.0001**	I	41	6	35	0.1698
II	56	27	29		II	51	5	46	
III	53	28	25		III	40	9	31	
IV	38	35	3		IV	38	10	28	

^a^For Cohort 1 protein analysis, the average patient age and tumor size was 66.7 years and 40.8 mm, respectively.

^b^The cut-off values for high/low ezrin expression were 2.0 for protein and 0.71for mRNA.

^c^For Cohort 2 mRNA analysis, the average patient age and tumor size was 67.3 years and 44.6mm, respectively.

^d^Statistically significant associations are shown in bold (*P* < 0.05).

### High ezrin expression predicts LN metastasis in CRC patients

To determine the predictive value of ezrin protein and mRNA expression in CRC, we performed univariate and multivariate logistic analysis of various factors and LN involvement (Table 2). In Cohort 1, the following clinicopathological factors were significantly related to LN metastasis in univariate logistic analysis: large tumor size (*P* = 0.0004), advanced T stage (*P* < 0.0001), poor differentiation/mucinous (*P* = 0.0115), lymphatic invasion (*P* < 0.0001), venous invasion (*P* < 0.0001), and high ezrin protein expression in CRC (*P* = 0.0085). Moreover, multivariate logistic analysis revealed that lymphatic invasion (odds ratio [OR] 14.6851, 95% confidence intervals [CI] 4.7346–64.9051; *P* < 0.0001) and high ezrin expression (OR 2.3088, 95% CI 1.1513–4.7120; *P* = 0.0183) were independent predictors of LN metastasis in CRC patients (Table 2). In Cohort 2, the following factors were significantly related to LN metastasis in univariate logistic analysis: advanced T stage (*P* < 0.0001), lymphatic invasion (*P* = 0.0023), venous invasion (*P* < 0.0001), and high ezrin expression in CRC (*P* = 0.0451). Additionally, multivariate logistic analysis revealed that advanced T stage (OR 3.0077, 95% CI 1.1436–8.9108; *P* = 0.0249) and high ezrin expression (OR 2.5128, 95% CI 1.0573–6.1922; *P* = 0.0369) were independent predictors of LN metastasis in CRC patients (Table 2).

**Table 2:** Univariate and multivariate analyses of associations with CRC with LN metastasis (logistic regression model).

Cohort 1: protein analysis^a^	Univariate analysis			Multivariate analysis		
Variables	OR	95% CI	*P* value	OR	95% CI	*P* value
Age (≥67 vs. <67 years)	1.009	0.5708-1.7858	0.9750	-	-	-
Gender (female vs. male)	1.4765	0.8302-2.6343	0.1846	-	-	-
Tumor size (≥41 vs. <41 mm)	2.8722	1.6013-5.2229	**0.0004**^b^	1.9018	0.9069-4.0139	0.0888
T classification (T3,4 vs. T1,2)	3.7754	1.9878-7.4742	**<0.0001**	1.2865	0.5369-3.0795	0.5699
Pathology (poor or mucinous vs. mod/well differentiated)	4.1643	1.3640-15.4991	**0.0115**	1.4951	0.4417-5.9988	0.529
Lymphatic invasion (present vs. absent)	22.4481	7.7765-95.2478	**<0.0001**	14.6851	4.7346-64.9051	**<0.0001**
Venous invasion (present vs. absent)	3.696	2.0040-6.9515	**<0.0001**	1.574	0.7425-3.3472	0.2359
Ezrin expression (≥2 vs. <2)^c^	2.1923	1.2191-4.0077	**0.0085**	2.3088	1.1513-4.7120	**0.0183**

Cohort 2: mRNA analysis^d^	Univariate analysis			Multivariate analysis		
Variables	OR	95% CI	p-value	OR	95% CI	p-value
Age (≥67 vs. <67 years)	1.145	0.6091–2.1487	0.6722	-	-	-
Sex (female vs. male)	1.1907	0.6328–2.2359	0.5869	-	-	-
Tumor size (≥45 vs. <45 mm)	1.5547	0.8328–2.9218	0.166	-	-	-
T classification (T3,4 vs. T1,2)	4.9697	2.1795–12.8905	**<0.00001**	3.0077	1.1436–8.9108	**0.0249**
Pathology (poor or mucinous vs. mod/well differentiated)	1.0668	0.3099–3.3512	0.9134	-	-	-
Lymphatic invasion (present vs. absent)	6.5852	1.8213–42.2762	**0.0023**	1.2427	0.1792–10.5130	0.8245
Venous invasion (present vs. absent)	6.5778	2.4450–22.9773	**<0.0001**	3.2869	0.9108–16.3262	0.0706
Ezrin expression (≥0.71 vs. <0.71)^c^	2.2614	1.0179–5.0840	**0.0451**	2.5128	1.0573–6.1922	**0.0369**

^a^For Cohort 1 protein analysis, the average patient age and tumor size was 66.7 years and 40.8 mm, respectively.

^b^Statistically significant associations are shown in bold (P < 0.05).

^c^The cut-off values for high/low ezrin expression were 2.0 for protein and 0.71 for mRNA.

^d^For Cohort 2 mRNA analysis, the average patient age and tumor size was 67.3 years and 44.6 mm, respectively.

### High ezrin protein and mRNA expression affects prognosis in CRC

**Figure 3:**
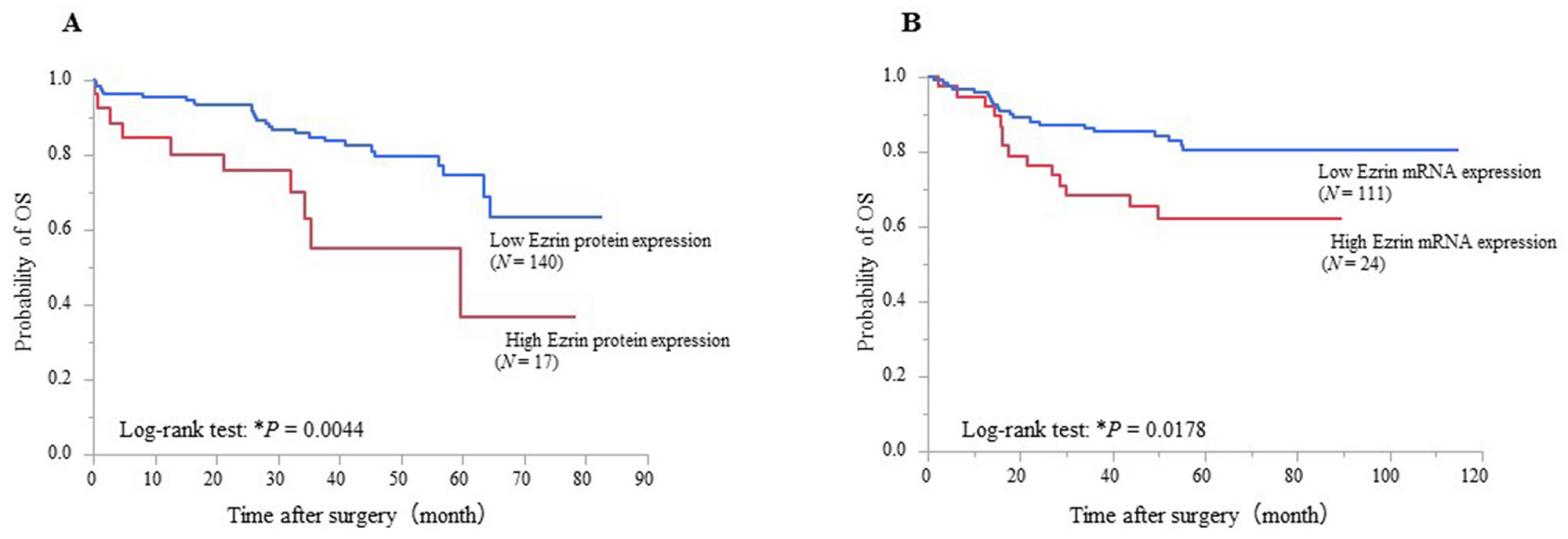
Survival curves of CRC patients after curative surgery according to their ezrin expression status **(A)** CRC patients (*N* = 157) were stratified by ezrin protein expression prior to curative surgery. Patients with high preoperative expression had poorer OS than those with low expression (*P* = 0.0044, log-rank test; cutoff value 5). **(B)** CRC patients (*N* = 135) were stratified by ezrin mRNA expression prior to curative surgery. Patients with high preoperative expression had poorer OS than those with low expression (*P* = 0.0178, log-rank test; cutoff value 0.58).

The results of univariate and multivariate logistic analysis of various factors and overall survival (OS) of CRC patients are shown in Table 3. Cohorts 1 and 2 were each divided into two groups of high and low ezrin expression using cutoff values determined by ROC analysis for prognosis (Cohort 1 = 5, Cohort 2 = 0.58). Kaplan–Meier analysis of Cohort 1 showed that OS was significantly poorer in the high ezrin expression group than the low expression group (*P* = 0.0044, log-rank test; Figure 3A). Univariate analysis showed that poor OS was significantly associated with large tumor size (*P* = 0.0217), advanced T stage (*P* = 0.0004), LN metastasis (*P* = 0.0008), distant metastasis (*P* = 0.0001), poor differentiation/mucinous (*P* = 0.0018), venous invasion (*P* = 0.0013), and high ezrin protein expression in CRC (*P* = 0.0124). Furthermore, multivariate analysis identified advanced T stage (Hazard Ratio [HR] ( 3.5129, 95% CI 1.2079–127563; *P* = 0.0198), distant metastasis (HR 5.5591, 95% CI 2.2504–13.6501; *P* = 0.0003), poor differentiation/mucinous (HR 2.9620, 95% CI 1.1080–7.0463; *P* = 0.0319), and high ezrin protein expression (HR 2.3125, 95% CI 1.0214–4.8337; *P* = 0.0447) were independent prognostic factors for CRC patients (Table 3). Kaplan–Meier analysis of Cohort 2 showed significantly poorer OS in the group with high ezrin mRNA expression than in the group with low expression (*P* = 0.0178, log-rank test; Figure 3B). Univariate analysis identified significant associations between poor OS and advanced age (*P* = 0.0007), large tumor size (*P* = 0.0035), advanced T stage (*P* < 0.0001), LN metastasis (*P* < 0.0001), distant metastasis (*P* < 0.0001), lymphatic invasion (*P* = 0.0062), venous invasion (*P* = 0.0054), and high ezrin mRNA expression in CRC (*P* = 0.0262). Additionally, multivariate analysis identified advanced T stage (HR 4.5510, 95% CI 2.1800–5.1080; *P* = 0.0001) and distant metastasis (HR 3.8089, 95% CI 1.7901–7.8652; *P* = 0.0008) as independent prognostic factors for CRC patients (Table 3).

**Table 3:** Univariate and multivariate analysis of associations with overall survival of CRC patients (Cox proportional hazards regression model).

Cohort 1: protein analysis^a^	Univariate analysis			Multivariate analysis		
Variables	HR	95% CI	*P* value	HR	95% CI	*P* value
Age (≥67 vs. <67 years)	1.0221	0.5392–1.9513	0.9464	-	-	-
Sex (female vs. male)	1.1388	0.5899–2.1703	0.6940	-	-	-
Tumor size (≥41 vs. <41 mm)	2.1199	0.2450–0.8953	**0.0217**^b^	1.2909	0.5778–2.9105	0.5320
T classification (T3,4 vs. Tis,1,2)	4.2072	1.7953–12.3012	**0.0004**	3.5129	1.2079–12.7563	**0.0198**
Lymph node metastasis	3.0386	1.5791–6.1326	**0.0008**	1.8401	0.9048–3.8839	0.0927
Distant metastasis	5.0544	2.3611–10.1594	**0.0001**	5.5591	2.2504–13.6501	**0.0003**
Pathology (poor or mucinous vs. mod/well differentiated)	4.7735	1.9072–10.4208	**0.0018**	2.962	1.1080–7.0463	**0.0319**
Lymphatic invasion (present vs. absent)	1.7639	0.7896–4.6936	0.1765	-	-	-
Venous invasion (present vs. absent)	2.8751	1.5170–5.5277	**0.0013**	1.2484	0.6071–2.6074	0.5470
Ezrin expression (≥5 vs. <5)^c^	2.7459	1.2651–5.4878	**0.0124**	2.3125	1.0214–4.8337	**0.0447**

Cohort 2: mRNA analysis^d^	Univariate analysis			Multivariate analysis		
Variables	HR	95% CI	*P* value	HR	95% CI	*P* value
Age (≥67 vs. <67 years)	2.7073	1.5141–5.0460	**0.0007**	2.0244	0.9946–4.2722	0.0518
Sex (female vs. male)	1.0514	0.5306–1.6711	0.8629	-	-	-
Tumor size (≥45 vs. <45 mm)	2.3455	1.3196–4.3201	**0.0035**	1.3162	0.6415–2.6436	0.4465
T classification (T3,4 vs. Tis,1,2)	6.3684	0.0963–10.3835	**<0.0001**	4.5510	2.1800–5.1080	**0.0001**
Lymph node metastasis	3.4421	1.9323–6.3528	**<0.0001**	1.4954	0.7406–3.0657	0.2611
Distant metastasis	9.6295	5.4338–17.0310	**<0.0001**	3.8089	1.7901–7.8652	**0.0008**
Pathology (poor or mucinous vs. mod/well differentiated)	1.9778	0.8594–3.9946	0.1027	-	-	-
Lymphatic invasion (present vs. absent)	7.0374	1.5422–124.5554	**0.0062**	1.7671	0.3181–33.2918	0.5694
Venous invasion (present vs. absent)	3.1039	1.3538–8.9684	**0.0054**	1.2428	0.1962–4.3652	0.7784
Ezrin expression (≥0.58 vs. <0.58)^c^	2.2183	1.1032–4.3253	**0.0262**	1.6608	0.7984–3.3463	0.1702

^a^For Cohort 1 protein analysis, the average patient age and tumor size was 66.7 years and 40.8 mm, respectively.

^b^Statistically significant associations are shown in bold (*P* < 0.05).

^c^The cut-off values for high/low Ezrin expression were 5.0 for protein and 0.58 for mRNA.

^d^For Cohort 2 mRNA analysis, the average patient age and tumor size was 67.3 years and 44.6 mm, respectively.

### Ezrin expression in CRC Cells

We next investigated the expression of ezrin in human CRC cell lines (Caco2, DLD1, HT29, LoVo, and SW480) using real-time PCR (Supplementary Figure 5A–5B). DLD1 showed the highest ezrin expression level (5.9-fold higher than the lowest expressing SW480 cells) followed by LoVo (4.2-fold), Caco2 (2.4-fold), and HT29 (2.2-fold) (Supplementary Figure 5B). To enable assessment of ezrin function in CRC cells, we performed siRNA-mediated ezrin knockdown in DLD1 and LoVo cells, which expressed the highest levels of ezrin. At 48 h after transfection, ezrin siRNA reduced gene expression up to 81% and 90% in DLD1 and LoVo cells, respectively, compared with the negative control siRNA (Supplementary Figure 5C–5D).

### Ezrin contributes to the migration and invasion capacity of CRC cells

**Figure 4:**
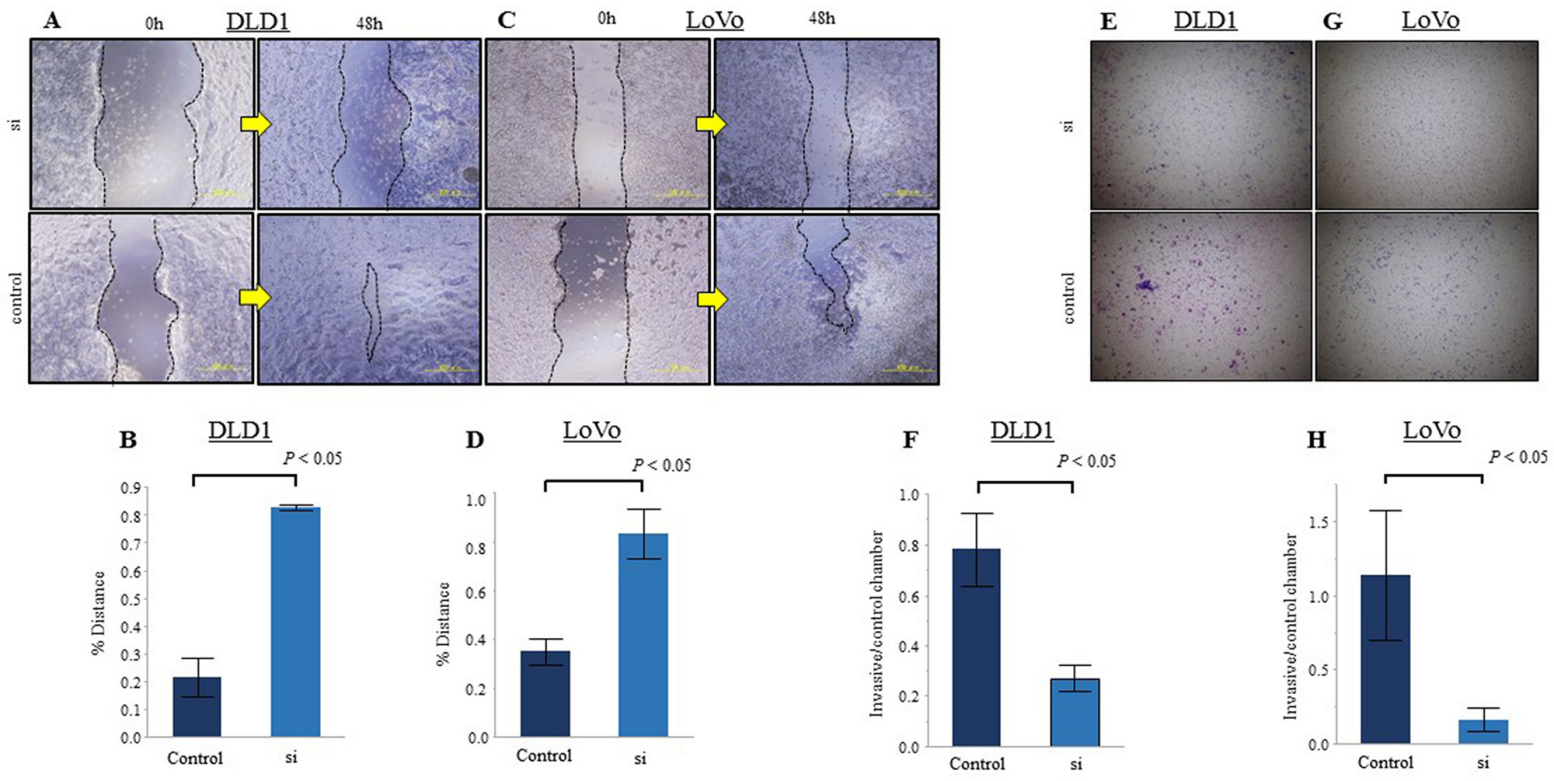
Reduction of ezrin expression inhibits CRC cell migration and invasion *in vitro* **(A, C)** Wound healing assays of DLD1 (A) and LoVo (C) CRC cells transfected with control or ezrin-specific siRNA. Representative images were acquired at 0 h and 48 h after wounding. **(B, D)** Quantitation of migration in the assays represented in A and C. The *y*-axis represents migration rates relative to control cells. **(E, G)** Transwell invasion assays of DLD1 (E) and LoVo (G) cells transfected with control or ezrin-specific siRNA. Phase contrast micrographs at ×40 magnification. **(F, H)** Quantitation of invasion in the assays represented in E and G. The *y*-axis represents the ratio of the number of invading cells per control chamber cells. All assays were replicated and the results are presented as the mean ± SEM.

We studied the cellular function of ezrin on migration and invasion by examining DLD1 and LoVo cells at 48 h after transfection with ezrin-targeting or negative control siRNA. We first performed wound healing assays to examine tumor cell motility and found that the migratory ability of both DLD1 and LoVo cells was significantly reduced by transfection with ezrin siRNA compared with control siRNA (Figure 4A–4D). Similar results were obtained in invasion assays, which showed that ezrin knockdown significantly decreased the invasiveness of DLD1 and LoVo cells compared with control cells (Figure 4E–4H).

## DISCUSSION

In this study, we performed iTRAQ-based proteomic analysis to identify a novel biomarker for CRC with LN metastasis. Of the 55 proteins differentially expressed in CRC specimens, we selected ezrin as the most promising candidate biomarker for predicting CRCs with LN metastasis. To validate the proteomics analysis, we investigated the pattern of ezrin expression in CRC tissues by IHC and real-time PCR. We found that levels of ezrin protein and mRNA were both significantly higher in CRC tissues than in adjacent normal colonic mucosa, and the expression increased significantly with increasing TNM stages. High expression of ezrin in CRC was significantly associated with tumor progression, as indicated by LN and distant metastases, and this observation was supported by our *in vitro* study showing that ezrin promotes the metastatic capacity of CRC cells by enabling cell invasion and migration. In multivariate analysis, high expression of ezrin protein and mRNA in CRC were independent predictive biomarkers for CRC with LN metastasis. Moreover, high ezrin protein expression was an independent prognostic factor in CRC patients.

Previous proteomics studies have sought to identify candidate proteins specifically expressed in CRC by comparing protein expression in CRC and normal colonic mucosa. Furthermore, previous searches for biomarkers associated with LN metastasis in CRC have mainly used 2-dimensional electrophoresis, which is a semi-quantitative method of assessing protein expression [14, 15]. By contrast, our study using the iTRAQ proteomics approach is the first comprehensive quantitative analysis of altered protein expression specifically associated with LN metastasis in CRC. We believe that our method is an easier and more precise approach to identifying novel candidate proteins predictive of LN metastasis in CRC.

Ezrin is a member of the ezrin–radixin–moesin (ERM) family of proteins, which link the actin-containing cytoskeleton to plasma membrane proteins and activate the actin cytoskeleton [16]. Ezrin binds to cell surface glycoproteins such as CD43, CD44, ICAM-1, and ICAM-2 through its amino-terminal domain and to filamentous actin through its carboxy-terminal domain [17]. Some of the signaling pathway components reported to be associated with ezrin function are protein kinase C, Rho kinase, NF-κB, and phosphatidylinositol 3-kinase/Akt [18]. A systemic review and meta-analysis of ezrin function revealed it to be an important signaling molecule associated with many cellular processes, including proliferation, adhesion, and motility, all of which play vital roles in tumorigenesis, invasion, and metastasis in a variety of human malignancies [19].

In normal colonic tissue, ezrin is located at the cell–cell adhesion region and is weakly present in the basal portion of epithelium, whereas in CRC cells, it is also present in the cytoplasm. High expression of ezrin protein correlates with the metastatic potential of several cancers, including prostatic intraepithelial neoplasia [20], osteosarcoma [21], rhabdomyosarcoma [22], and CRC [23]. However, ours is the first multivariate analysis to demonstrate a correlation between ezrin levels and LN metastasis in CRC. In our study, high ezrin expression was significantly associated with highly metastatic behaviors such as LN and distant metastasis. Ezrin activation and subsequent interaction with membrane proteins and the cytoskeleton may promote cell migration, invasion, adhesion, and survival, which are important for cancer progression [24]. Ohtani et al. reported that ezrin transcription was necessary for invasion and consequent tumor progression [25], and consistent with this, our *in vitro* experiments clearly demonstrate that knockdown of ezrin significantly reduces migration and invasion of CRC cells. It is possible that ezrin might influence the assembly of cytoskeletal elements at the cytoplasmic face of the plasma membrane and thus facilitate cell migration and invasion. These results are in agreement with reports that changes in the cytoskeleton might be a key factor regulating neoplastic progression and tumor growth. Suppression of ezrin expression and disruption of its significantly reduced lung metastasis in a mouse osteosarcoma model [26]. In addition, a recent study indicated that ezrin binds to another actin-binding protein, cortactin, in cancer cells to promote formation of podosomal rosettes, which digest underlying fibronectin and promote invasion [27]. Meng et al. suggested that ezrin might play functional roles in promoting the morphology, growth, motility, and invasion of pancreatic cancer cells and, further, that the Erk1/2 pathway may be involved in these roles [28-30]. These observations suggest that ezrin is an important factor for metastatic potential in CRC and is a novel target to prevent further metastasis from primary sites.

Previous reports have linked overexpression of ezrin and prognosis in various cancers, including ovarian carcinoma [31], hepatitis B-related hepatocellular carcinoma [32], non-small-cell lung cancer [33], and CRC [34-36]. In our study, patients in the high ezrin expression group had significantly poorer prognosis than those in the low-expression group; indeed, high ezrin expression was shown to be an independent prognostic marker in these patients. Interestingly, we found that ezrin protein and mRNA expression were both independent predictors of LN metastasis in CRC patients. Because real-time PCR analysis of mRNA levels is more quantitative and clinically informative than protein intensity scoring by IHC, it could potentially be exploited for preoperative evaluation. Jin et al. reported that ezrin mRNA levels were increased in non-small cell lung cancer specimens compared with adjacent non-tumor tissues and normal tissues [37]. In addition, Ogino et al. reported that levels of ezrin mRNA in osteosarcoma samples were higher in patients with lung metastasis than in those without metastasis [38]. However, there have been no previous quantitative evaluations of ezrin levels and their relationship to the clinicopathological features in CRC. In this study, ezrin protein and mRNA expression were both independent predictors of LN metastasis in CRC patients. Thus quantification of ezrin mRNA expression in preoperative biopsy samples could be a useful tool for predicting metastasis in CRC patients. Therefore, in clinic, when we take biopsy samples from CRC patients by endoscopy, and evaluate the expression levels of ezrin preoperatively, we are able to evaluate the risk of LN metastases in CRC patients, and to decide the strategy of treatment, preoperatively. Currently, several surgical options are available for patients with early stage CRC, including endoscopic mucosal resection, submucosal dissection, and surgical negative with regional lymphadenectomy. The level of ezrin detected in preoperative biopsies could facilitate therapeutic decision-making. Thus, if LN metastasis is not predicted, we can select a suitable endoscopic treatment and minimal surgical procedure. In contrast, if LN metastasis is predicted, management with neoadjuvant and/or adjuvant chemotherapy might be preferred.

In conclusion, our systematic proteomics approach has identified high expression of not only ezrin protein but also ezrin mRNA as novel predictive biomarkers of LN metastasis in CRC. To the best of our knowledge, this is the first report of such an association, and quantification of ezrin mRNA levels in biopsy samples preoperatively may thus be a valuable tool for planning the treatment of CRC patients. For clinical application, however, further prospective studies are needed to validate our preliminary results using preoperative biopsy samples from CRC patients.

## MATERIALS AND METHODS

### Patients, study design, and sample collection

Patients with primary CRC who underwent surgical resection at the Mie University Medical Hospital, Japan, between January 2000 and 2011 were enrolled in this study. All samples were collected from resected specimens. Our study analyzed 646 surgical samples comprising of 20 samples as the initial iTRAQ discovery set, and 261 normal and 365 CRC samples as the validation set.

A summary of the workflow for this study is shown in Figure 1. A discovery set of 20 samples was used for the iTRAQ analysis and consisted of CRC patients with LN metastasis (N = 5, stage III), CRC without LN metastasis (N = 5, stage II), adjacent normal mucosa from CRC patients (N = 5), and normal colonic mucosa from benign colorectal disease (N = 5). The pathological T classification of all CRC samples in this study is SS stage. In addition, we prepared 45 samples of CRC with LN metastasis (N = 14, stage III), CRC without LN metastasis (N = 14, stage I–II), and normal colonic mucosa (N = 17) for analysis of mRNA expression of the top four candidate proteins by real-time PCR.

For the validation study, we enrolled two specimen cohorts for expression analysis of ezrin protein and mRNA. Cohort 1 consisted of 195 high quality formalin-fixed paraffin-embedded (FFPE) tissues from resected primary cancer specimens obtained between 2006 and 2011, which were analyzed for ezrin protein expression by immunohistochemistry (IHC). Cohort 2 consisted of 170 fresh-frozen CRC samples collected between 2000 and 2006, which were analyzed for ezrin gene expression by real-time PCR. Additional details of the patients are provided in the Supplementary Materials and Methods.

### Cell lines

Human CRC cell lines Caco2, DLD1, HT29, LoVo, and SW480 were obtained from the Cell Resource Center of Biomedical Research Institute of Development, Aging and Cancer (Tohoku University, Sendai, Japan). CRC cell lines were maintained in RPMI1640 medium supplemented with 10% Fetal Bovine Serum, 100 IU/mL penicillin, and 100 μg/mL streptomycin and were maintained at 37°C in a humidified 5% CO2 atmosphere. The authenticity of the cell lines was routinely monitored by analyzing DNA (short tandem repeat) specific for each cell line in an approved laboratory (last tested on July 15, 2014). siRNA transfection protocols are described in the Supplementary Materials and Methods.

### iTRAQ proteomics analysis

All steps for iTRAQ, including protein preparation (extraction, precipitation, assay, reduction, alkylation, and trypsin digestion, iTRAQ labeling, LC-MS/MS), and data analysis are detailed in the Supplementary Materials and Methods.

### Quantitative real-time PCR

Total RNA was isolated from the surgical specimens or CRC cell lines using an RNeasy Mini Kit (Qiagen, Valencia, CA, USA) and the cDNA was synthesized with random hexamers and Superscript III reverse transcriptase (Invitrogen) according to the manufacturer’s instructions. Target gene expression was analyzed by real-time PCR using Power SYBR Green PCR Master Mix (Applied Biosystems, Carlsbad, CA, USA) and a Step One Plus Real-Time PCR system (Applied Biosystems). Ezrin mRNA levels in each sample were normalized to β-actin mRNA levels using the 2-ΔCT method. Additional details are provided in the Supplementary Materials and Methods.

### Immunohistochemistry

Ezrin IHC was performed on FFPE tissue sections (3 μm thick). Immunoreactivity scores were assigned based on the combined intensity and extent of ezrin staining (Supplementary Figure 1). Further information is provided in the Supplementary Materials and Methods.

### Wound healing and invasion assays

Wound healing and invasion assays to assess the function of ezrin were performed on DLD1 and LoVo human CRC cells transfected with control or ezrin-specific siRNA. Experimental details are provided in the Supplementary Materials and Methods.

### Statistical analysis

All statistical analyses were performed using JMP version 10 (SAS Institute, Cary, NC). The results are expressed as the median values or as the mean ± SE. Comparisons were performed using the non-parametric Mann–Whitney U-test for continuous variables. Differences between groups were estimated using Pearson’s χ2 test or Kruskal–Wallis test. Receiver operating characteristic (ROC) curves were established for determining cutoff values of protein and mRNA expression for prediction of LN metastasis and prognosis using Youden’s index. The Kaplan–Meier method was used to determine the cumulative probability of overall survival and the differences were evaluated using log-rank tests. Prognostic factors were examined by univariate and multivariate analysis (Cox proportional hazards regression model). Logistic regression analysis was used to evaluate the factors influencing LN metastasis. P values less than 0.05 were considered statistically significant.

## SUPPLEMENTARY MATERIALS FIGURES AND TABLE





## Abbreviations

CRC: colorectal cancer
CT: computed tomography
ERM: ezrin–radixin–moesin
FFPE: formalin-fixed paraffin-embedded
HR: hazard ratio
IHC: immunohistochemistry
iTRAQ: isobaric tags for relative and absolute quantitation
LC: liquid chromatography
LN: lymph node
MRI: magnetic resonance imaging
MS: mass spectrometry
OS: overall survival
PANTHER: Protein Analysis through Evolutionary Relationship
ROC: receiver operating characteristic
