# Supplementary material for: Successful identification of a predictive biomarker for lymph node metastasis in colorectal cancer using a proteomic approach
Source: Oncotarget. 2017 Oct 30;8(63):106935–47. doi: 10.18632/oncotarget.22149 (PMC5739786; doi:10.18632/oncotarget.22149)
Supplement: Supplementary file 1 [file oncotarget-08-106935-s001.pdf]

Successful identification of a predictive biomarker for lymph node metastasis in colorectal cancer using a proteomic approach

SUPPLEMENTARY MATERIALS

Patients, study design, and sample collection

Clinicopathological findings were based on The Union for International Cancer Control tumor node metastasis (TNM) classification. Cohort 1 comprised of 113 men and 82 women (average age, 66.7 years), of whom 48, 56, 52, and 38 patients had stage I, II, III, and IV CRC, respectively. The median follow-up time was 33.6 months (range 1–83 months). Cohort 2 comprised of 99 men and 71 women (average age, 67.3 years), of whom 41, 51, 40, and 38 patients had stage I, II, III, and IV CRC, respectively. The median follow-up time was 52.4 months (range 1–115 months). No patient received chemotherapy or radiotherapy before surgery and no perioperative mortalities were observed. Exclusion criteria included inflammatory bowel disease, familial adenomatous polyposis, hereditary non-polyposis colon cancer, or other rare and complex types of tumors.

Protein extraction and preparation for iTRAQ

Frozen samples were weighed (6–20 mg) and homogenized with TissueLyser II (QIAGEN). Samples were kept frozen on dry ice and then lysed by addition of a buffer (50 mM ammonium bicarbonate, 50 mM dithiothreitol, 0.5% sodium deoxycholate [SDC]) containing a protease inhibitor cocktail. The samples were then sonicated on ice by 20 pulses of 10 s each using an Ultrasonic Disruptor (TOMY). Samples were centrifuged for 10 min at 130,000 ×*g*. The supernatants were removed, mixed with 5 volumes of acetone (stored at −20°C), and incubated overnight at −20°C. The precipitates were centrifuged for 15 min at 16,000 ×*g*. Protein pellets were air dried and then resuspended in 0.5 M triethylammonium bicarbonate (TEAB) containing 0.5% SDC. Finally, the protein concentration of each sample was determined using a bicinchoninic acid assay (Pierce, Rockford, IL Cat# 23227). Protein extracts were aliquoted and stored at −80°C until further analysis.

iTRAQ labeling

A total of 100µg of protein per group was used for iTRAQ labeling. TEAB and SDC were added to each sample to a final concentration of 0.5 M and 0.5%, respectively. Proteins were reduced and alkylated according to the iTRAQ kit manufacturer's instructions (AB SCIEX, Foster City, CA). Samples were digested overnight at 37°C with trypsin (sequencing grade modified, Promega) at a ratio of 1:30. Samples were labeled with iTRAQ 4-plex reagents (Applied Biosystems, Foster City, CA). First, we labeled 20 samples individual as follows: CRC with LN metastasis, iTRAQ reagent 114; CRC without LN metastasis, iTRAQ reagent 117; CRC adjacent normal mucosa, iTRAQ reagent 115; and normal colonic mucosa, iTRAQ reagent 116. Second, we mixed the four different surgical labeled samples, including CRC with LN, CRC without LN, CRC adjacent normal mucosa and normal colonic mucosae as one group. Third, we mixed the four samples and incubated for 1 h at room temperature, and made five groups as same method. The individual one group mixed Interfering substances was removed using a cation-exchange cartridge system (AB SCIEX P/N 4326747) according to the manufacturer's instructions. The ion-exchange eluate was desalted using Sep-Pak C18 cartridges (Agilent, Palo Alto, CA). Finally, peptides in each sample were analyzed using a QTRAP5500 LC-MS/MS system (AB SCIEX) and data analysis was performed using ProteinPilot software (AB SCIEX) (Figure 1).

iTRAQ-Sample Clean-up Prior to LC-MS/MS Analysis

Before performing LC-MS/MS analysis, the iTRAQ-labeled peptides were cleaned up using cation-exchange cartridge system (P/N 4326747, SCIEX, Redwood City, CA) to remove unbound iTRAQ tags and salts. The combined iTRAQ-labeled peptides were diluted at least 10 fold with Cation Exchange Buffer (CEB)-Load (10 mM potassium phosphate (KH2PO4) in 25% (v/v) acetonitrile, pH 3.0) and the pH was adjusted with phosphoric acid to 2.5~3.3. Injection of sample mixture into the strong-cation exchange (SCX) cartridge (4.0 mm × 15 mm) containing POROS® 50 HS, 50µm particles was done manually using a sterile 2.5 mL syringe at the rate of approximately 1 drop per second and the flow-through was collected. After inject 1ml of CEB-Load to wash the TCEP, SDS, calcium chloride, and excess iTRAQ Reagents from the cartridge, bound iTRAQ-labeled peptides were eluted by slowly injecting 500 µL of CEB–Elute (10 mM KH2PO4 in 25 % (v/v) acetonitrile containing 350 mM potassium chloride (KCl), pH 3.0) and the elute was collected (Sample elution 1-6). Subsequently, the collected elute was diluted with 0.1% formic acid (FA) prior to passing through Sep-Pak C18 cartridges (Agilent, Palo Alto, CA) for desalting. By injecting 1.5ml buffer (70% acetonitrile (ACN), 0.1% FA), the flow-through was collected. Then, he solution was

speed-vac to dryness, and re-constituted in 50uL with 0.1 FA, and stored at -20°C before LC-MS/MS analysis.

Liquid chromatography-electrospray ionization mass spectrometry

Mass spectrometric analysis (MS) of these samples was performed using a QTRAP 5500 triple quadrupole linear ion trap mass spectrometer (SCIEX, Framingham, MA, USA) coupled with DiNa nanoLC autoinjection system (KYA Technologies Corporation, Tokyo, Japan) for acquisition of MS and tandem MS (MS/MS) data. Peptides were loaded on a HiQ Sil C18W-3 column (10 cm×100μm id) (Particle Size : 3μm, Pore Size :120 Å; KYA Technologies Corporation, Tokyo, Japan) and then submitted to mobile-phase elution using 2% acetonitrile containing 0.1% formic acid (mobile phase A) and 70% acetonitrile containing 0.1% formic acid (mobile phase B). The peptides were eluted at a flow rate of 300 μL/min. Gradient conditions were: 0% B (0 to 10 min), 5–100% B (70 min), 100% B held for 10 min, 0% B (10 min).

The liquid chromatography eluent was directed to an electrospray ionization source for scanning linear ion trap. The QTRAP 5500 system was operated in enhanced mass scan triggered information dependent acquisition (EMS-Triggered IDA) mode using Analyst 1.5 software (SCIEX, Framingham, MA, USA). Electrospray ionization (ESI) was performed for IDA in the positive-ion mode with a spray voltage of 2300 V, curtain gas (CUR), 10.00, ion source gas1 (GS1), 15.00, ion source gas 2 (GS2), 0.00, collision gas (CAD), high, interface heater temperature (IHT), 150°C, entrance potential (EP), 10.00, eQ1 and Q3, unit resolution with scan rate of 10000 Da/s. All the samples were consisted with that mentioned with the iTRAQ-based shotgun analysis. In IDA analysis, after each survey scan of the m/z range from 400 to 1000 and an enhanced resolution scan, the three highest-intensity ions with multiple charge states were selected for MS/MS using a rolling collision energy based on the ions observed charge states and masses.

Proteomics Data Analysis

The acquired raw data files were processed by Protein Pilot software 4.0 (revision 148085; SCIEX, Framingham, MA, USA) using the Paragon algorithm 4.000, revision 148083 for the peptide identification. The three triplicate LC-MS/MS injections were combined for database searching against the UniProtKB/Swiss-Prot Homo sapiens proteome database (June 2008; 276834 sequences).

Quantitative real-time PCR

The primers for ezrin amplification were: forward, CCGGGAAGTGTGGTACTTTG and reverse, GGGATTCTCCTTCCTGACCT. The primers for β-actin, which was amplified as an internal control, were: forward, ACAGAGCCTCGCCTTTGC and reverse, GCGGCGATATCATCATCC. Amplification conditions were 95°C for 10 min, 40 cycles of 95°C for 15 s and 60°C for 1 min. After amplification, the products were subjected to an increasing temperature gradient from 60°C to 95°C at a rate of 0.3°C/s with continuous fluorescence monitoring to produce a melting curve. All reactions were performed in duplicate.

Immunohistochemistry

Formalin-fixed paraffin-embedded tissue sections (3 μm) were prepared from surgical specimens of patients with CRC. After deparaffinization and dehydration, the sections were placed in 10 mM sodium citrate buffer (pH 6.0) and autoclaved at 121°C for 10 min for antigen retrieval. The sections were incubated in 3% hydrogen peroxide for 10 min to block endogenous peroxidase activity and then incubated in normal goat serum (Vector Laboratories Inc., Burlingame, CA) for 1 h to block nonspecific binding. The sections were then incubated with a primary antibody to ezrin (Abcam ab40839, 1:500 dilution) for 1 h at room temperature. Antibody binding was visualized using EnVision reagents (Dako REAL EnVision Detection System; peroxidase/DAB+, Dako Cytomation, Denmark). All sections were counterstained with hematoxylin-eosin prior to dehydration and mounting.

Immunohistochemistry scoring

Each slide was observed by scanning the entire tissue specimen under low-power (×40) and high-power (×100) magnification. Two independent pathologists with no prior knowledge of the clinicopathological parameters evaluated ezrin immunoreactivity at the core of the CRC sample based on the intensity and extent of staining. The staining intensity was scored as follows: 0 = no staining, 1 = weak staining, 2 = moderate staining, and 3 = strong staining (Supplementary Figure 1A-1D). The staining extent was scored according to the percentage of CRC cells with cytoplasmic ezrin expression as follows: 0 = 0%, 1 = 1–25%, 2 = 26–50%, 3 = 51–75%, and 4 = 76–100% (Supplementary Figure 1E-1H). Finally, the immunochemistry scores were calculated by multiplying the intensity and extent scores, resulting in a scale with a minimum score of 0 and a maximum score of 12. Any discrepancies encountered were resolved using a multihead microscope until a consensus was reached.

Ezrin siRNA interference

Ezrin-specific small interfering RNA (siRNA) (Silencer Predesigned siRNA) and negative control siRNA

(Silencer Negative Control siRNA) were purchased from Ambion (Austin, TX). Cancer cell lines were seeded at 2 × 10^5 cells per well in a final volume of 2 mL in 6-well flat-bottomed microtiter plates. Cells were cultured overnight to allow them to adhere. siRNAs were diluted with Opti-MEM I Reduced Serum Medium (Invitrogen, Carlsbad, CA), mixed with diluted Lipofectamine RNAiMAX Reagent (Invitrogen), and incubated for 5 min at room temperature to allow formation of siRNA–Lipofectamine RNAiMAX Reagent complexes. The complexes were then added to the wells at a final concentration of 10 nM. Cells were incubated at 37°C in a humidified 5% CO_2 atmosphere.

Wound healing assay

Transfected CRC cells were incubated in a 6-well plate and allowed to form a confluent monolayer. An artificial wound was generated in the cell monolayer with a sterile 200 µL pipette tip and the culture medium was replaced to remove detached cells. Wound closure was assessed 48 h later using an Olympus IX71 microscope (Olympus, Center Valley, PA) at ×40 magnification. Images of cells from the same field were acquired at the indicated time points using Adobe Photoshop 9.0.2 software and compared with baseline measurements. Each measurement was performed in triplicate.

Invasion assay

Invasion assays were performed using 24-well (8 µm pore size) BioCoat Matrigel Invasion Chambers and 24-well (8 µm pore size) BioCoat Control Inserts (SD Biosciences Discovery Labware, Franklin Lakes, NJ). Cells were transfected with control or ezrin siRNA as described above; 24 h later, they were seeded in the top chamber at 5 × 10^4 cells per 500 µL in serum-free RPMI medium. The top chambers were placed into a BD Falcon TC Companion Plate filled with 20% FBS-containing medium, and the chambers were incubated at 37°C in a humidified 5% CO_2 atmosphere. After 48 h incubation, the medium and non-invading cells in the top chamber were removed with a cotton swab and Phosphate Buffered Saline. To quantify the invading cells on the bottom side of the membranes, the membranes were fixed in methanol, stained with Diff-Quik stain (Sysmex Corp., Kobe, Japan), and mounted on glass slides. The cells were counted in five microscopic fields using a light microscope at ×40 magnification. Each experiment was performed in triplicate.

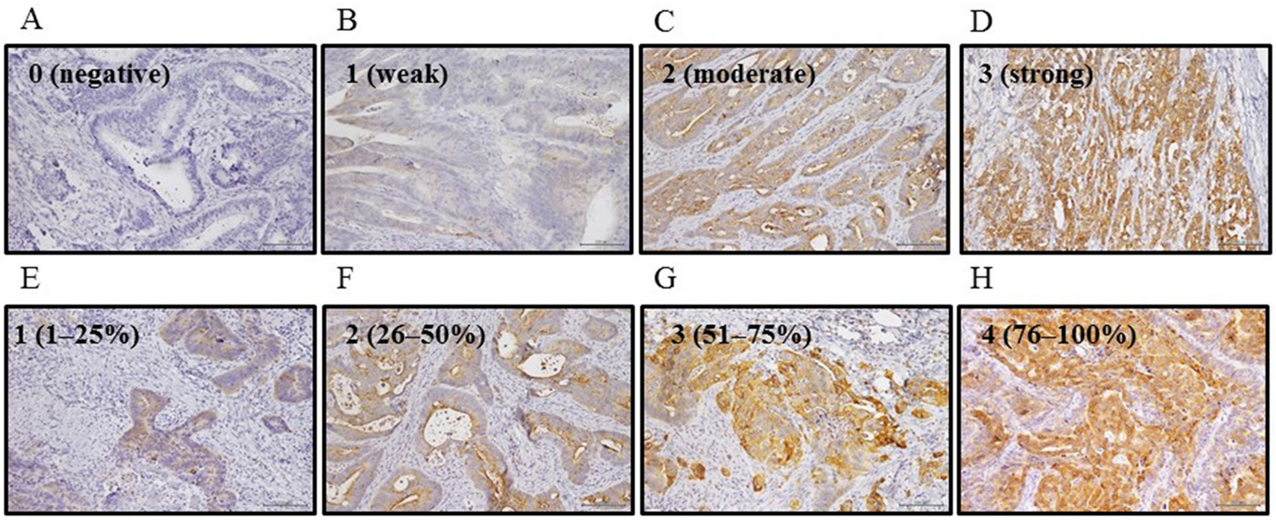

Supplementary Figure 1: Scoring system for immunohistochemical analysis of ezrin expression in CRC. (A-D) The intensity of ezrin protein staining was scored as negative = 0 (A), weak = 1 (B), moderate = 2 (C), and strong = 3 (D). **(E-H)** The extent of ezrin protein staining was scored as the percentage of cancer cells with cytoplasmic staining: 0 = 0%, 1 = 1–25% (E), 2 = 25–50% (F), 3 = 51–75% (G), and 4 = 76–100% (H). Representative images are shown. All images were captured at ×100 original magnification.

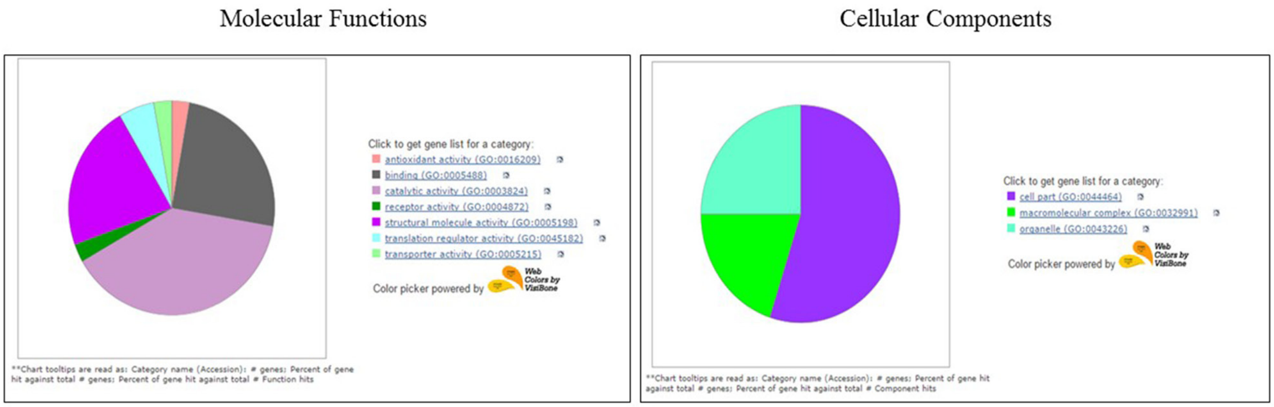

Supplementary Figure 2: Bioinformatics analysis of classification for "Molecular Functions" and "Cellular Components" terms for the 55 differentially expressed proteins in CRC (PANTHER Classification System, version 11. 1, analyzed in April 2017).

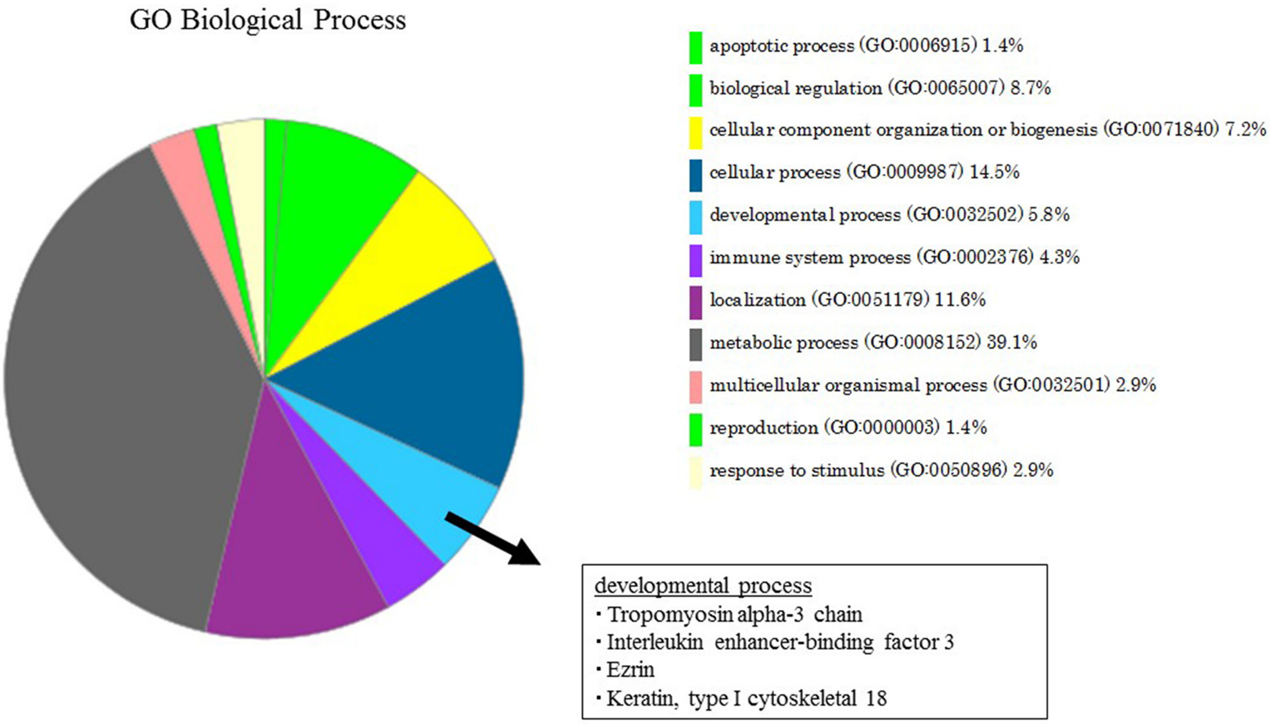

Supplementary Figure 3: Bioinformatics analysis of biological process terms for the 55 differentially expressed proteins in CRC (PANTHER Classification System, version 10. 0, analyzed in December 2015). Tropomyosin alpha-3 chain, interleukin enhancer-binding factor 3, ezrin, and keratin, type I cytoskeletal 18 were selected as proteins annotated to the "developmental process."

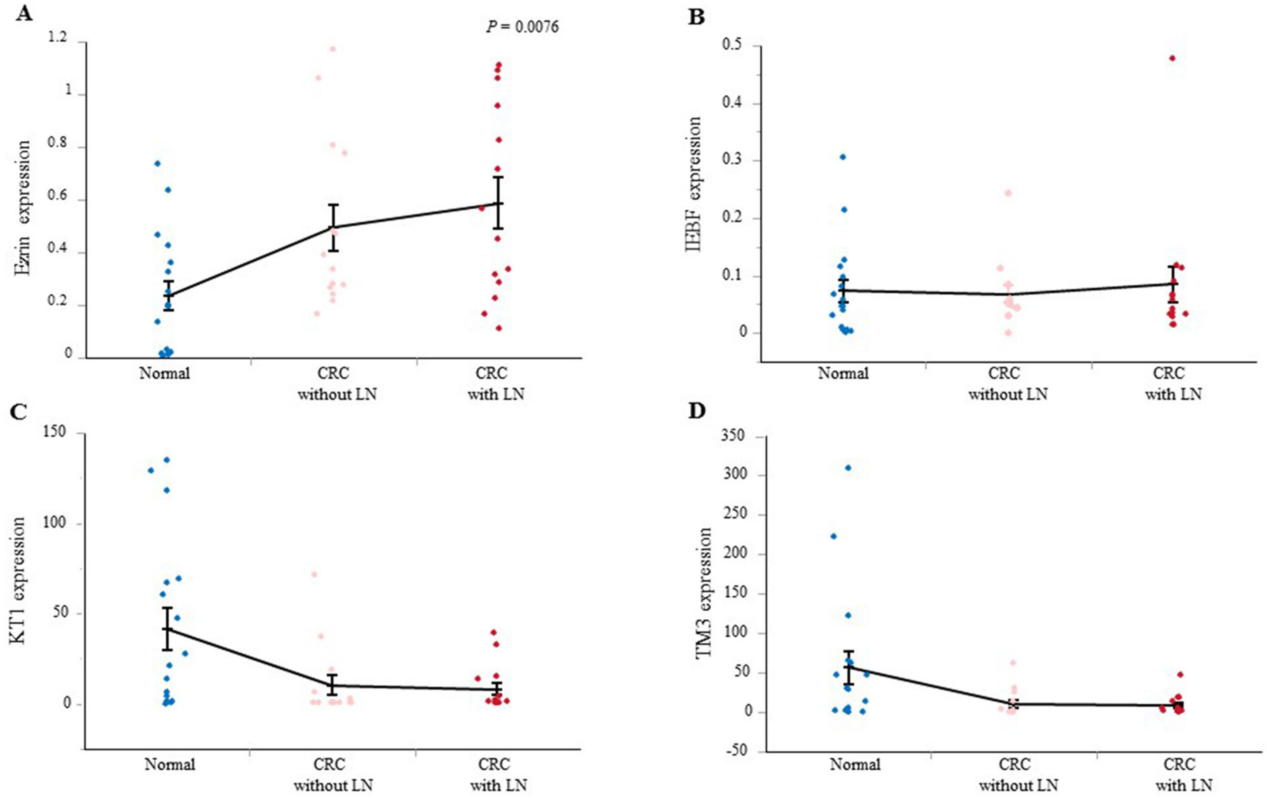

Supplementary Figure 4: mRNA expression of candidate marker proteins selected from the screening phase. (A-D) Subsets of specimens from normal mucosa (N = 17), CRC without LN metastasis (N = 14), and CRC with LN metastasis (N = 14) were analyzed by real-time PCR to quantify ezrin (A), interleukin enhancer-binding factor 3 (B), keratin, type I cytoskeletal 18 (C), and tropomyosin alpha-3 chain (D) mRNA levels. Data are the mean ± SEM, with median values indicated by the central horizontal line. Statistical analysis was performed using the Kruskal–Wallis test.

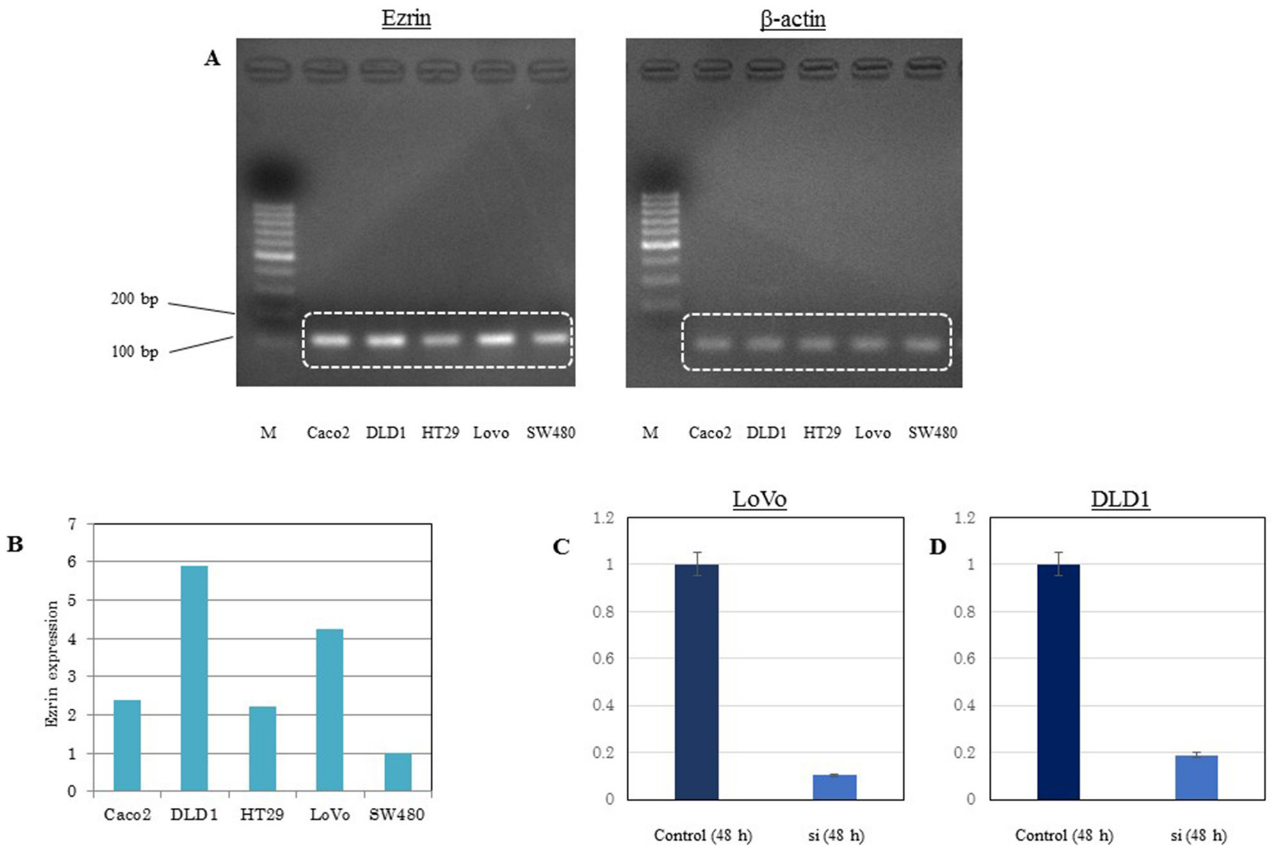

Supplementary Figure 5: Expression of ezrin mRNA in CRC cell lines and the effect of siRNA transfection. **(A)** Real-time PCR analysis of ezrin and β-actin mRNA expression in five CRC cell lines. M indicates the bp marker lane. **(B)** Quantification of relative ezrin mRNA expression in five CRC cell lines. **(C, D)** Expression of ezrin mRNA in DLD1 (C) and LoVo (D) at 48 h after transfection with control or ezrin-specific siRNA.

Supplementary Table 1: Differentially expressed proteins in CRC with lymph node metastasis identified by iTRAQ analysis.

See Supplementary File 1
